# Effect of 3D Printing Parameters on the Viscoelastic Behavior of Acrylonitrile Butadiene Styrene: Fractional Calculus Modeling and Statistical Optimization

**DOI:** 10.3390/polym17121650

**Published:** 2025-06-13

**Authors:** Flor Y. Rentería-Baltiérrez, Jesús G. Puente-Córdova, Juan M. Hernández-Ramos, Arlethe Y. Aguilar-Villarreal, Nasser Mohamed-Noriega

**Affiliations:** 1 Faculty of Chemical Sciences, Universidad Autónoma de Nuevo León, Pedro de Alba s/n, San Nicolás de los Garza 66455, Nuevo León, Mexico; flor.renteriabltz@uanl.edu.mx (F.Y.R.-B.); arlethe.aguilarvll@uanl.edu.mx (A.Y.A.-V.); 2 Faculty of Mechanical and Electrical Engineering, Universidad Autónoma de Nuevo León, Pedro de Alba s/n, San Nicolás de los Garza 66455, Nuevo León, Mexico; nasser.mohamednr@uanl.edu.mx

**Keywords:** 3D printing, fractional Zener model, response surface methodology, Taguchi method, viscoelasticity

## Abstract

This study addresses the challenge of optimizing the viscoelastic performance of acrylonitrile butadiene styrene (ABS) parts manufactured by fused deposition modeling (FDM), where printing parameters strongly influence mechanical properties. The objective was to systematically evaluate the effects of four key factors—infill pattern, build orientation, layer height, and filament color—on storage modulus, damping factor, and glass transition temperature. A combined experimental design approach was employed: Taguchi’s L9 orthogonal array efficiently screened parameter effects, while response surface methodology (RSM) enabled detailed analysis of interaction effects and multiresponse optimization. Results revealed that build orientation and layer height had the greatest impact, increasing instantaneous stiffness ($E_{u}$) by up to 81%, equilibrium modulus ($E_{0}$) by 128%, and glass transition temperature ($T_{g}$) by 1.46%, while decreasing the damping factor (tan δ) by 3.4% between optimized and suboptimal conditions. To complement the statistical optimization, the fractional Zener model (FZM) was applied to characterize the viscoelastic response of two representative samples optimized for either high stiffness or high flexibility. The flexible sample exhibited a higher fractional order (α=0.24), indicating enhanced elastic mobility, while the stiff sample showed a higher activation energy ($E_{a}=0.52$ eV), consistent with restricted molecular motion. This integrated approach provides a robust and generalizable framework for improving material performance in polymer additive manufacturing.

## 1. Introduction

Additive manufacturing (AM), commonly known as 3D printing, has seen widespread adoption across various industries, including construction, prototyping, and biomechanics. Although this technology was first introduced by Charles Hull in 1986 [1], its rapid advancement in recent years has expanded its applications significantly. AM enables the fabrication of complex three-dimensional structures that would be difficult or costly to produce using conventional manufacturing methods [2,3]. One of the most widely used AM techniques is fused deposition modeling (FDM), in which a polymer filament is heated above its glass transition temperature and extruded through a nozzle, layer by layer, to create a final object [4]. Among AM processes, FDM stands out for manufacturing thermoplastic components due to its advantages, including low initial investment, minimal material waste, ease of material change, and the availability of commercial 3D printing software [5]. This process is particularly well suited for producing low-stiffness polymer components efficiently.

Despite its advantages, fully understanding the FDM process remains a challenge, as the mechanical properties of printed specimens are influenced by numerous parameters. Key factors include infill pattern, infill density, layer thickness and width, air gaps within or between layers, extrusion speed and temperature, raster angle, build orientation, and nozzle diameter [6,7,8,9]. Furthermore, FDM-printed components exhibit anisotropic behavior due to the orientation of the infill patterns, which resembles the fiber alignment in polymer composites [10,11,12,13].

The development of new products through AM involves critical design and manufacturing decisions that significantly impact the performance of the final product. Key considerations include recyclability, risk minimization, sustainability, manufacturability, material selection, durability, assembly, cost, and maintenance. One of the main challenges in AM is ensuring that printed components meet requirements for geometry, functionality, and cost while maintaining the reliability of the production system. In this context, FDM has become one of the most widely used techniques for processing a variety of thermoplastic polymers, including acrylonitrile butadiene styrene (ABS), polylactic acid (PLA), polycarbonate (PC), polystyrene (PS), thermoplastic polyurethane (TPU), and polyethylene terephthalate (PET) [14,15]. However, because FDM components are constructed by successive layer deposition, weak interlayer adhesion often leads to mechanical properties inferior to those of injection-molded parts [16]. Consequently, optimizing the printing parameters is essential to improve the mechanical performance, dimensional accuracy, and overall quality of 3D printed materials.

Although factors such as infill pattern, layer thickness, raster angle, and build orientation are commonly studied, the influence of color and pigment additives remains less explored. Previous studies have shown that additives can affect the crystallinity, tensile strength, and even extrusion temperature of polymers [17,18].

Research on the impact of filament color in FDM-printed materials remains limited and often inconsistent, with most studies focusing on static rather than dynamic mechanical properties. For instance, Sung-Hoon et al. [12] reported minimal impact of color on the tensile strength of ABS parts, while Gao et al. [18] found significant differences in mechanical properties among various colored ABS and PLA samples. Similarly, Alves et al. [13] observed that white ABS exhibited superior tensile strength compared to other colors, suggesting that the pigment composition—often undisclosed by filament manufacturers—can play a crucial role in mechanical performance. Building on this growing body of evidence that filament pigmentation can affect the structural and thermal behavior of printed parts, it is noteworthy that color remains an understudied parameter in additive manufacturing. This oversight is especially relevant given that pigment additives may influence molecular mobility, heat absorption, and polymerization dynamics, which can alter mechanical behavior under dynamic conditions. Although filament color is frequently treated as a purely aesthetic attribute, a deeper understanding of its functional implications could enhance the predictive accuracy of part performance, particularly in thermally demanding or load-bearing applications. However, current studies have primarily addressed static mechanical responses, leaving a gap in our understanding of how printing parameters and filament color influence the dynamic mechanical behavior of printed materials. Given that viscoelastic properties are essential for components subjected to cyclic loading, vibrations, or time-dependent deformation, evaluating the temperature-dependent response of different colored filaments under dynamic mechanical analysis (DMA) becomes crucial. This is particularly relevant in fields such as automotive engineering, where FDM-printed parts are exposed to mechanical and thermal cycling, which may accelerate material degradation and failure at elevated temperatures [19].

Dynamic mechanical analysis (DMA) is widely recognized as a fundamental technique for characterizing the viscoelastic behavior of polymers, especially in materials processed through AM. This method provides essential insights into storage modulus, loss modulus, and damping characteristics (tan δ), which are critical to evaluating the performance of FDM-printed parts under dynamic conditions. Additionally, DMA is one of the most reliable techniques for determining key thermal transitions, such as glass transition temperature ($T_{g}$), softening and melting points, damping capacity, and crystallization behavior [20].

Focusing on ABS, previous research has shown that its dynamic mechanical properties are highly influenced by process parameters such as layer thickness, infill density, nozzle temperature, infill pattern, layer height, and print orientation. For instance, He and Khan [5] reported the storage modulus under different printing parameter settings, where the flat orientation provided the highest storage modulus. They observed that the influence of layer thickness appeared to be insignificant, while the nozzle size had a significant influence on the storage modulus. Priya et al. [11] reported that ABS specimens exhibit higher $T_{g}$ values with the triangular infill pattern. Additionally, they observed a decrease in thermal properties (storage modulus, loss modulus, and $T_{g}$) with increasing layer height. Galeja et al. [21] analyzed the effect of raster angle and printing configuration, finding that a 55° raster angle resulted in the highest impact strength despite increased porosity. They also emphasized the brittleness of FDM-printed ABS due to poor interlayer adhesion, reinforcing that FDM cannot yet match injection molding in mechanical performance.

Despite advances in optimizing static mechanical properties, there is limited exploration of its viscoelastic behavior under dynamic conditions. Addressing these gaps through comprehensive DMA studies will deepen the understanding of how FDM process parameters influence viscoelastic behavior, guiding the optimization of 3D printing conditions for enhanced mechanical performance. Additionally, this study aims to explore how filament color, in combination with factors such as infill pattern, layer height, and print orientation, can further influence the dynamic mechanical response, particularly in terms of temperature-dependent behavior.

To improve the mechanical performance of FDM-printed parts, it is crucial to optimize the process parameters using robust statistical techniques. Design of experiments (DOE) provides a systematic approach to assess the influence of multiple variables simultaneously, allowing for more efficient parameter selection and performance prediction. Several DOE methodologies have been applied in AM, including full factorial designs, Taguchi method, and the response surface methodology (RSM), among others [3,5]. Taguchi methods are particularly useful for minimizing variability and identifying optimal parameter settings using orthogonal arrays [7,8], while ANOVA (analysis of variance) helps determine which factors significantly impact mechanical properties [22,23]. RSM, on the other hand, models complex relationships between multiple parameters, enabling a more refined optimization process [6]. In recent studies, a combination of DOE, Taguchi, and RSM has been successfully implemented to optimize mechanical properties in hybrid composite materials, including polymer matrices reinforced with natural and synthetic fibers [24].

This study investigates the temperature-dependent viscoelastic behavior of ABS components fabricated via FDM, considering the combined influence of infill pattern, layer height, build orientation, and filament color. To address the multifactorial nature of these influences, a novel integration of three complementary approaches was employed: the Taguchi method for efficient screening of optimal parameter levels, response surface methodology (RSM) for predicting multiresponse behavior, and fractional-calculus-based modeling using the fractional Zener model (FZM) to characterize the mechanical response under dynamic loading. This hybrid strategy bridges the gap between statistical process optimization and constitutive material modeling, offering both practical guidelines for refining FDM processing conditions and theoretical insights into the dynamic performance of ABS under real-world operating conditions. By incorporating these tools, the study not only provides a robust framework for optimizing the mechanical performance of 3D-printed polymers but also reveals novel insights into how color-dependent properties influence viscoelastic behavior across a temperature sweep.

## 2. Materials and Methods

### 2.1. Printed Samples

The material used in this study was acrylonitrile butadiene styrene (ABS) copolymer in three different colors: white, black, and magenta. These colors represent different pigmentation in ABS filaments and were used to evaluate whether color influences the mechanical response of printed specimens under identical processing conditions. The filament, supplied in proprietary cartridges for the CubePro Trio 3D printer (3D Systems, model 40173, Rock Hill, SC, USA), had a nominal diameter of 1.75 mm. According to the supplier’s safety data, the polymer content ranges between 95 and 100%. The ABS filament was used as received, without any chemical or physical modifications. Molecular weight information indicating a high molecular weight ($M_{W}∼$ 150,000 g/mol) was obtained from the technical datasheet provided by 3D Systems. According to manufacturer specifications, the recommended extrusion temperature for this ABS ranges from 220 °C to 260 °C. The material exhibits a density of approximately 1.05 g/cm^3^ and is known for its high impact resistance, even at low temperatures, as well as excellent resistance to creep under sustained load. The computer-aided design (CAD) of the specimens was created using Creo Parametric 2.0 software (PTC Inc., Boston, MA, USA) with a rectangular geometry of 50 mm in length, 7 mm in width, and 3 mm in thickness. These dimensions were selected to evaluate the optimal conditions of the 3D printing process while ensuring compatibility with the mechanical testing setup. A total of 81 specimens were printed and tested. The fixed printing parameters, summarized in Table 1, were kept constant throughout the process in accordance with the manufacturer’s recommendations to ensure sample quality, dimensional accuracy, and reproducibility. These guidelines specify which settings must remain fixed to maintain print stability and quality and which parameters can be varied to study their influence on the mechanical performance of the printed specimens.

**Figure 1:**
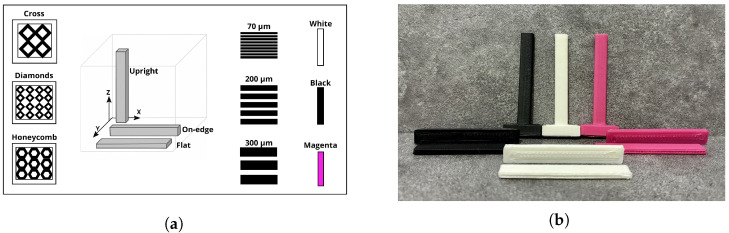
(**a**) Schematic representation of the four printing parameters: infill pattern, build orientation, layer height, and color. (**b**) Photographs of 3D-printed specimens used in this study.

The objective of this study was to assess the effect of selected design and process variables on the mechanical behavior of 3D-printed ABS specimens. Four key parameters were considered: infill pattern, build orientation, layer height, and color (Figure 1a). These parameters influence not only the mechanical properties but also the overall efficiency of the fused deposition modeling (FDM) process in terms of manufacturing time and cost. Among the selected variables, one was quantitative (ABS layer height), while the remaining three were qualitative. The next step in setting up the design of experiments (DOE) was to determine the experimental resolution and the number of levels for each variable. To complement the experimental design, Figure 1b presents a photograph of the printed specimens used in this study. This visual representation highlights the diversity of test conditions related to color, build orientation, and geometric configuration, and serves to illustrate the physical consistency of the samples analyzed during the viscoelastic characterization.

### 2.2. Dynamic Mechanical Analysis

Dynamic mechanical analysis (DMA) was performed to characterize the viscoelastic response of the samples under a low-amplitude sinusoidal deformation. The printed specimens were tested in single cantilever mode using a dynamic mechanical analyzer (DMA 8000, PerkinElmer Inc., Waltham, MA, USA). A temperature sweep was conducted from 293 K to 473 K at a heating rate of 2 K/min, with a fixed displacement of 0.01 mm and a frequency of 1 Hz. This analysis provided valuable insights into the thermomechanical behavior of ABS under controlled dynamic loading conditions, allowing for a comprehensive evaluation of its viscoelastic performance. Once representative samples were selected for fractional modeling, additional multifrequency DMA tests were performed at 0.01, 0.1, 1, and 10 Hz. These measurements enabled the determination of the parameters of the FZM directly from experimental data, using Arrhenius-type plots to characterize the temperature-dependent response. To ensure the reliability of the experimental data, all tests were conducted under controlled laboratory conditions using calibrated equipment. A total of 81 DMA specimens were printed and tested according to the full factorial design. Additionally, selected conditions were replicated to verify the reproducibility of the results. As the DMA involved temperature-sweep tests, responses were not averaged across replicates; instead, consistency was assessed by comparing key parameters such as storage modulus and glass transition temperature. The accuracy of the DMA system is ±1% for both force and displacement, as specified by the manufacturer, and temperature was controlled via a built-in thermocouple with a precision of ±0.1 K. Statistical methods, including MANOVA and Taguchi analysis, were employed to evaluate the robustness and consistency of the results.

### 2.3. Design of Experiment

A design of experiment (DOE) approach was implemented to systematically plan and structure laboratory experiments, ensuring statistically reliable and reproducible results. Table 2 presents the two main components of DOE, factors and levels, organized in an experimental matrix. Statistical analyses were performed using Minitab 19 (Minitab LLC, State College, PA, USA) and Python (version 3.13.1, Python Software Foundation, Wilmington, DE, USA). Minitab was used for the design and execution of the experimental matrix. Python was used for complementary statistical analyzes, data visualization, and validation of the results, ensuring reproducibility and flexibility in data processing. This allowed a quantitative evaluation of the effects of the selected parameters on the mechanical properties of the printed specimens, specifically storage modulus at low and high temperatures (293 K and 473 K), loss factor, and glass transition temperature. The printed samples were further analyzed using both multivariate analysis of variance (MANOVA) and response surface methodology (RSM) to evaluate response performance and suitability. The selection of MANOVA was justified by the significant correlation found among the four response variables, as assessed using Pearson’s correlation test. Finally, the effects were analyzed using an orthogonal array design based on the Taguchi methodology, optimizing the number of experiments without compromising the quality of the analysis. This combination of statistical methods allowed for a robust assessment of the studied variables and provided a deeper understanding of their influence on the mechanical behavior of 3D-printed ABS specimens.

### 2.4. Fractional Viscoelastic Model

**Figure 2:**
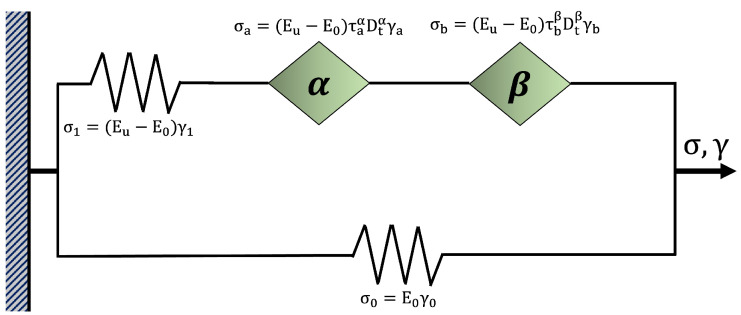
The FZM with two spring-pots, α and β. The fractional calculus formalism follows the Riemann–Liouville definition.

The viscoelastic response of the 3D-printed samples was described using the fractional Zener model (FZM), which extends the classical Zener model by incorporating fractional calculus formalism. This model uses two spring-pot elements (Figure 2), which interpolate between purely elastic (Hookean) and purely viscous (Newtonian) behavior, enabling a more accurate representation of molecular mobility within the printed polymeric structures [25,26].

Each spring-pot is defined by a power-law relationship characterized by a fractional order parameter (α or β) and a characteristic relaxation time (τ). These parameters capture the cooperative molecular dynamics at different temporal scales: α accounts for short relaxation times (low temperatures, high frequencies), while β describes long relaxation times (high temperatures, low frequencies) [27,28].

The constitutive equation of the FZM used is:

(1) $$\left(E_{u}-E_{0}\right)\gamma =\left(\sigma -E_{0}\gamma \right)+\tau_{\beta }^{-\beta }D_{t}^{-\beta }\left(\sigma -E_{0}\gamma \right)+\tau_{\alpha }^{-\alpha }D_{t}^{-\alpha }\left(\sigma -E_{0}\gamma \right)$$

where $E_{u}$ and $E_{0}$ are the unrelaxed and relaxed moduli, respectively, and $D_{t}^{-\alpha }$, $D_{t}^{-\beta }$ represent the Riemann–Liouville fractional integrals describing viscoelastic behavior at different timescales [29,30]. Considering that the ABS material evaluated through DMA is subjected to a sinusoidal mechanical input, the complex modulus $E^{*}=E^{\prime }+iE^{\prime \prime }$ can be formulated as a function of the angular frequency ω at a fixed temperature by means of Fourier transform techniques, as described in previous studies [31]. Based on this approach, Equation (2) describes the resulting expression for $E^{*}$, which was obtained from the general form presented in Equation (1). Furthermore, by separating the real and imaginary components of Equation (2), the storage modulus $E^{\prime }$ and the loss modulus $E^{\prime \prime }$ were derived, as presented in Equations (3) and (4), respectively. Additionally, the damping behavior of the material, represented by the loss factor (tan δ), can be directly calculated from the ratio $\frac{E^{\prime \prime }}{E^{\prime }}$.

(2) $$E^{*}\left(i\omega \right)=E^{\prime }+iE^{\prime \prime }=\frac{E_{u}+E_{0}\left[{\left(i\omega \tau_{a}\right)}^{-\alpha }+{\left(i\omega \tau_{b}\right)}^{-\beta }\right]}{1+{\left(i\omega \tau_{a}\right)}^{-\alpha }+{\left(i\omega \tau_{b}\right)}^{-\beta }}$$

(3) $$E^{\prime }=E_{0}+\frac{\left(E_{u}-E_{0}\right)\left[1+{\left[\omega \tau_{a}\right]}^{-\alpha }\cos\left(\alpha \frac{\pi }{2}\right)+{\left[\omega \tau_{b}\right]}^{-\beta }\cos\left(\beta \frac{\pi }{2}\right)\right]}{{\left[1+{\left[\omega \tau_{a}\right]}^{-\alpha }\cos\left(\alpha \frac{\pi }{2}\right)+{\left[\omega \tau_{b}\right]}^{-\beta }\cos\left(\beta \frac{\pi }{2}\right)\right]}^{2}+{\left[{\left[\omega \tau_{a}\right]}^{-\alpha }\sin\left(\alpha \frac{\pi }{2}\right)+{\left[\omega \tau_{b}\right]}^{-\beta }\sin\left(\beta \frac{\pi }{2}\right)\right]}^{2}}$$

(4) $$E^{\prime \prime }=\frac{\left(E_{0}-E_{u}\right)\left[{\left[\omega \tau_{a}\right]}^{-\alpha }\sin\left(\alpha \frac{\pi }{2}\right)+{\left[\omega \tau_{b}\right]}^{-\beta }\sin\left(\beta \frac{\pi }{2}\right)\right]}{{\left[1+{\left[\omega \tau_{a}\right]}^{-\alpha }\cos\left(\alpha \frac{\pi }{2}\right)+{\left[\omega \tau_{b}\right]}^{-\beta }\cos\left(\beta \frac{\pi }{2}\right)\right]}^{2}+{\left[{\left[\omega \tau_{a}\right]}^{-\alpha }\sin\left(\alpha \frac{\pi }{2}\right)+{\left[\omega \tau_{b}\right]}^{-\beta }\sin\left(\beta \frac{\pi }{2}\right)\right]}^{2}}$$

To account for temperature effects on the relaxation behavior, a cooperative model was employed based on an Arrhenius-type equation with a temperature-dependent cooperativity factor:

(5) $$\tau_{\mathrm{cooperative}}=\tau_{0}\exp\left(\frac{ZE_{a}}{k_{B}T}\right);\;Z\left(T\right)=\frac{T(T^{*}-T_{0})}{T^{(}T-T_{0})}$$

where $\tau_{0}$ is the pre-exponential factor, $E_{a}$ is the activation energy, $k_{B}$ is Boltzmann’s constant, and $T^{*}$, $T_{0}$ are characteristic temperatures [32,33]. This framework explains how relaxation times increase with cooperativity at low temperatures, requiring more energy for molecular rearrangements. The parameters α, β, $\tau_{0}$, and $E_{a}$ were obtained by fitting the model to dynamic mechanical analysis (DMA) data at multiple frequencies. The agreement between theoretical predictions and experimental curves confirms the validity of the FZM in capturing the viscoelastic behavior of 3D-printed ABS under various printing and thermal conditions.

## 3. Results and Discussion

### 3.1. Dynamic Mechanical Analysis

Dynamic mechanical analysis (DMA) was employed to investigate the viscoelastic properties of the 3D-printed ABS samples under varying temperature conditions. DMA is particularly useful in evaluating the transition from a glassy state to a rubbery state, assessing both the elastic (storage modulus, E′) and viscous (loss modulus, E″) components of the material. The elastic part represents the material’s ability to store energy, while the viscous part corresponds to the dissipation of energy.

**Figure 3:**
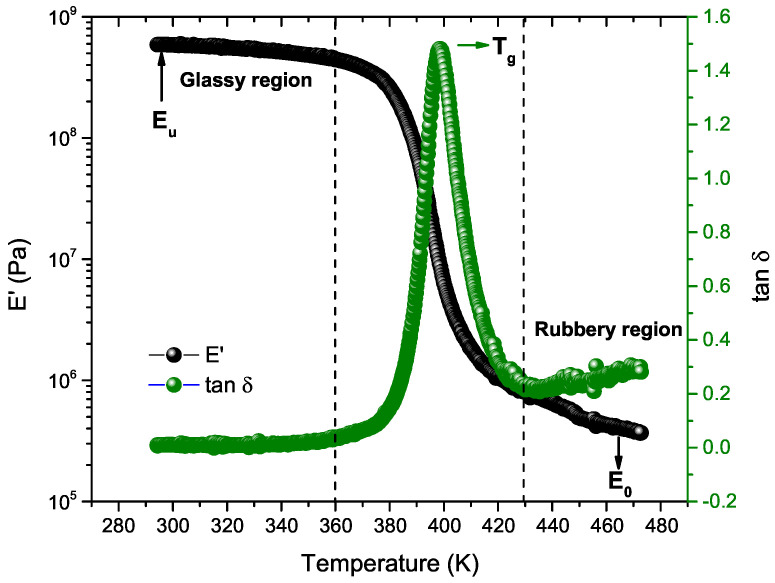
Storage modulus ($E^{\prime }$) and damping factor (tan δ) vs. temperature for a representative ABS sample.

Figure 3 presents the DMA results for a representative ABS sample, indicating a decrease in the storage modulus (E’) as the temperature increases. This decline reflects the material’s transition from a glassy, rigid state with limited molecular mobility to a more flexible, elastomeric state with increased chain mobility [19]. Regarding the tan δ (the ratio of the loss modulus to the storage modulus, tan δ = E”/E’), a peak is observed around 396 K, corresponding to the material’s $T_{g}$. This peak indicates maximum energy dissipation and is a critical point for evaluating the material’s damping capacity [20,34]. At temperatures above 430 K, tan δ shows a marked increase, signaling the material’s transition toward viscous flow. This behavior is particularly significant for ABS transformation processes, including injection molding, extrusion, and 3D printing, where understanding these mechanical and viscoelastic changes is crucial for optimizing processing conditions [14].

Table 3 summarizes the mean storage moduli at low and high temperatures ($E_{u}$ and $E_{0}$), the loss factor (tan δ), and the glass transition temperature ($T_{g}$) for different printing parameter combinations. The values correspond to a fractional subset of the full factorial design. As the statistical analysis progresses (e.g., through MANOVA, RSM, or Taguchi methods), additional samples are incorporated as required to support deeper interpretation of the results. This approach allowed for the assessment of the combined effects of the studied factors, such as infill pattern, build orientation, layer height, and color, on the viscoelastic properties of ABS. The results are discussed to highlight the significance and interactions of these parameters in shaping the material’s mechanical response.

### 3.2. Initial Data Exploration

**Figure 4:**
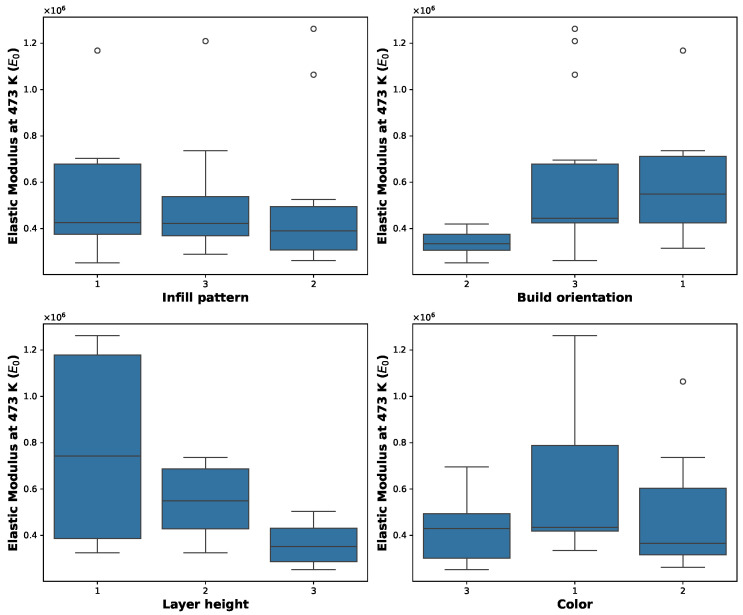
Boxplots of storage modulus at 473 K ($E_{0}$) grouped by infill pattern, build orientation, layer height, and filament color.

Before performing variance analysis techniques, an initial data exploration was performed to identify potential outliers and assess the distribution of the data. Verifying data normality is essential when applying analysis of bariance (ANOVA) to ensure valid statistical inferences or, if necessary, to apply suitable data transformations. Figure 4 presents boxplot visualizations for the analyzed responses, highlighting the presence of outliers. The response variable $E_{0}$ showed the most significant number of outliers across the factors of infill pattern, build orientation, and color. The rest of the response variables, $E_{u}$, tan δ, and $T_{g}$, exhibited fewer outliers for some of the factors considered. Based on these initial findings, a normality test was performed for each response, yielding the following *p*-values: $E_{u}$ (*p*-value = 0.5713), $E_{0}$ (*p*-value = 0.0000), tan δ (*p*-value = 0.3778), and $T_{g}$ (*p*-value = 0.7431). These results indicate that the $E_{0}$ response does not follow a normal distribution, requiring data transformation prior to conducting variance analysis [35].

**Figure 5:**
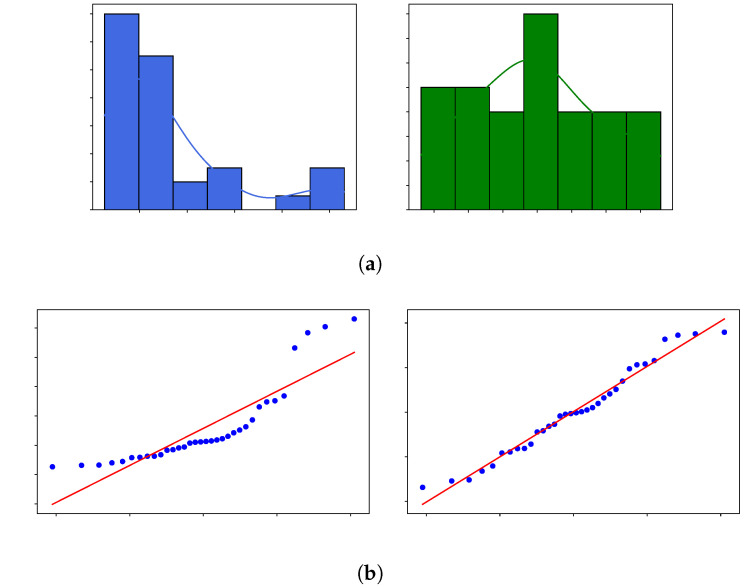
Histograms and Q-Q plots of elastic modulus at 473 K ($E_{0}$) before and after Box–Cox transformation. (**a**) Histograms with kernel density estimates (KDE) showing the distribution of $E_{0}$. (**b**) Q–Q plots comparing empirical quantiles to a normal distribution; red line indicates theoretical normality.

To address this issue, the Shapiro–Wilk test was applied, and a Box–Cox transformation was performed. After the transformation, the *p*-value for $E_{0}$ improved to 0.4134, with an optimal Box–Cox parameter of λ=−0.9428. Figure 5 displays the histograms and Q-Q plots before and after the data transformation, demonstrating that the transformed data did not reject the normality hypothesis. Consequently, the normalized dataset met the assumptions required for ANOVA, allowing for a more accurate and reliable interpretation of the results. The Box–Cox transformation was selected because it provides a systematic method to identify the most appropriate power transformation that stabilizes variance and improves data normality, essential for valid ANOVA results. Various λ values were tested to maximize the log-likelihood function, with the optimal value found to be λ=−0.9428. This value indicates a transformation close to the inverse of the variable, reflecting the original data’s deviation from normality. The improvement in normality was confirmed by the Shapiro–Wilk test, as the *p*-value increased from 0.0000 (non-normal) to 0.4134 (normal). Figure 5 illustrates these changes through histograms and Q–Q plots.

### 3.3. Multivariate Analysis of Variance (MANOVA)

Experimental data collection, analysis, and interpretation were performed following established statistical techniques for each model considered in this study, such as MANOVA, Taguchi, and RSM, to comprehensively assess the factors affecting the mechanical behavior of 3D-printed ABS. The performance of MANOVA is essential for a comparative evaluation of these statistical models, similar to previous analyses of the influence of factorial designs on the characterization of complex systems [36,37]. MANOVA was utilized to identify significant factors and their effects on the viscoelastic properties of the printed specimens. Given that the DMA tests measured multiple responses, storage modulus at low and high temperatures, loss factor, and glass transition temperature, MANOVA effectively evaluated the simultaneous influence of the experimental factors on these interrelated responses. This approach allowed for a comprehensive assessment of whether the factors had a global impact before applying more detailed models. According to the literature, denser infill patterns or higher resolutions generally enhance material stiffness, potentially increasing the storage modulus [16,38]. Similarly, the build orientation may contribute to material anisotropy, affecting the mechanical and viscoelastic properties [5]. This variability should be reflected in the dependent variables analyzed. Additionally, the influence of color on mechanical properties is plausible, as pigments can alter the polymer’s composition or thermal stability, leading to variations in storage modulus or $T_{g}$ depending on the selected colors [18]. Table 4 presents the MANOVA results, revealing that all four factors (infill pattern, build orientation, layer height, and color) have a significant global impact on the responses. The Wilks’ lambda, Pillai’s trace, Hotelling–Lawle trace, and Roy’s greatest root tests all resulted in statistically significant values (p<0.05), confirming the relevance of these factors. The individual *p*-value of each factor was notably low, indicating their significant influence on the multivariate responses. Notably, the factor color exhibited a slightly higher *p*-value (0.0605) in Roy’s greatest root test, suggesting it may be near the threshold of statistical significance. However, it was still considered relevant due to its potential impact on the material’s performance.

### 3.4. Analysis of Variance (ANOVA)

Based on the MANOVA results, ANOVA was applied to examine the specific effects of each manufacturing process parameter on the measured viscoelastic properties. ANOVA evaluates the variance between different groups to determine whether there are significant differences among the levels of each factor [22,23]. Typically, it is used to analyze the percentage of influence each parameter has on the output individually, as shown in Table 5. The Box–Cox transformation improved data normality, as evidenced by the Shapiro–Wilk test (*p* = 0.4134), indicating that the transformed data do not reject the null hypothesis of normality. According to the ANOVA and post hoc Tukey/Games–Howell tests, the following observations were made:

- Storage modulus at 293 K ($E_{u}$): The infill pattern showed a *p*-value of 0.7823, suggesting no statistically significant differences among the levels (1, 2, and 3). The Tukey HSD test supported this, with only the pair 1–2 showing a significant difference (*p* = 0.0253), while other comparisons had p> 0.05. Build orientation had a *p*-value of 0.3894, indicating no significant effects, and the Tukey test confirmed no significant differences between groups. Layer height exhibited a *p*-value of 0.000283, highlighting significant differences among groups. Tukey’s post hoc analysis showed significant differences between groups 1–3 and 2–3 (p< 0.05). For color, the Welch ANOVA did not show significant differences (*p* = 0.7396), supported by the Games–Howell test with all *p*-values above 0.05.
- Storage modulus at 473 K ($E_{0}$): Build orientation did not show significant differences (*p* = 0.6615); however, the Tukey test identified a significant difference between groups 2 and 3 (*p* = 0.0405), indicating a potential interaction. Layer height had a significant *p*-value (0.004682) in the Welch ANOVA, and the Games–Howell test showed significant differences between groups 2 and 3 (*p* = 0.0115). Color showed a *p*-value of 0.0428, suggesting potential significance, but the Tukey test did not reveal significant differences between any specific groups (p> 0.05).
- Loss factor (tan δ): The infill pattern had a *p*-value of 0.086975, which is near the significance threshold, suggesting a possible trend without reaching statistical significance. Build orientation did not show significant effects (*p* = 0.6889), though the Tukey test showed a significant difference between groups 2 and 3 (*p* = 0.0249). The remaining factors did not display significant differences across groups (*p* > 0.05).
- Glass transition temperature ($T_{g}$): Build orientation showed a significant *p*-value (0.0146), and the Tukey test confirmed significant differences between groups 1 and 3 (*p* = 0.0449). Color exhibited a *p*-value of 0.0554, which is close to the significance level, although no significant differences were found between the specific groups.

Overall, the significant differences identified by the ANOVA occurred primarily in layer height for $E_{u}$, layer height and color for $E_{0}$, and build orientation for $T_{g}$. Partial differences were observed for the infill pattern concerning tan δ and color for $T_{g}$. These findings suggest that the manufacturing parameters, particularly layer height and build orientation, have a notable influence on the viscoelastic properties of the 3D-printed ABS specimens. Consistent observations have been reported in the literature, where layer height was shown to critically affect void geometry, interlayer fusion, and energy absorption during impact, all of which influence the mechanical performance of 3D-printed parts [39]. Additionally, the influence of printing orientation observed in this study aligns with previous reports [5,9], which indicate that controlling the filament deposition angle can enhance the mechanical properties of 3D-printed components.

**Table 5:** ANOVA results and post hoc tests for each viscoelastic response variable.

Variable	Factor	*F* (ANOVA)	*p* (ANOVA)	Significative Groups
$E_{u}$	Infill pattern	0.0776	0.7823	1, 2
	Build orientation	0.7615	0.3894	–
	Layer height	16.6092	0.0003	1, 3; 2, 3
	Color	0.3068	0.7396	–
$E_{0}$	Infill pattern	0.0254	0.8743	–
	Build orientation	0.1953	0.6615	2, 3
	Layer height	8.5845	0.0047	2, 3
	Color	3.7582	0.0428	–
tan δ	Infill pattern	3.1179	0.0869	–
	Build orientation	0.1631	0.6889	2, 3
	Layer height	0.0181	0.8936	–
	Color	0.6366	0.4308	–
$T_{g}$	Infill pattern	0.3891	0.5372	–
	Build orientation	3.6147	0.0146	1, 3
	Layer height	0.4297	0.5168	–
	Color	2.3945	0.0554	–

### 3.5. Response Surface Methodology

**Figure 6:**
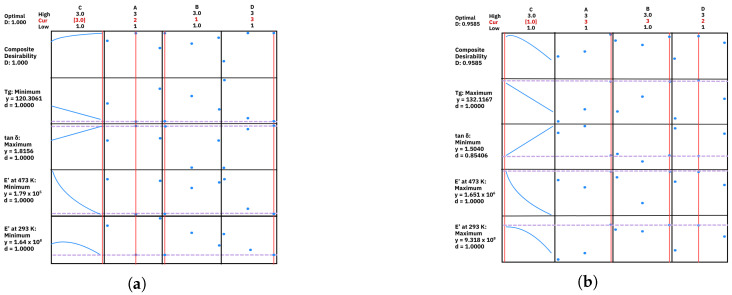
Multiresponse optimization using RSM: (**a**) conditions maximizing damping and minimizing stiffness, (**b**) conditions maximizing stiffness and minimizing damping.

Response surface methodology (RSM) is an empirical modeling technique used to investigate the relationships between dependent and independent variables [24,35]. In this study, RSM was applied to establish functional relationships between the printing parameters and the temperature-dependent dynamic mechanical properties. Two optimization approaches were considered to identify the optimal printing conditions. The first approach aimed to minimize $E_{u}$, $E_{0}$, and $T_{g}$ while maximizing tan δ. The results, shown in Figure 6a, indicate that the optimal combination for this scenario consists of a diamonds infill pattern, flat orientation, a layer height of 300 μm, and the magenta color. The second approach was designed for applications requiring the opposite effect: maximizing $E_{u}$, $E_{0}$, and $T_{g}$ while minimizing tan δ (Figure 6b). The optimal conditions for this scenario were found with a honeycomb infill pattern, edge orientation, a resolution of 70 μm, and black color. These findings provide valuable insights into selecting appropriate printing parameters for specific application requirements, optimizing the balance between stiffness and damping behavior in the 3D-printed ABS samples.

### 3.6. Taguchi Analysis Results

**Figure 7:**
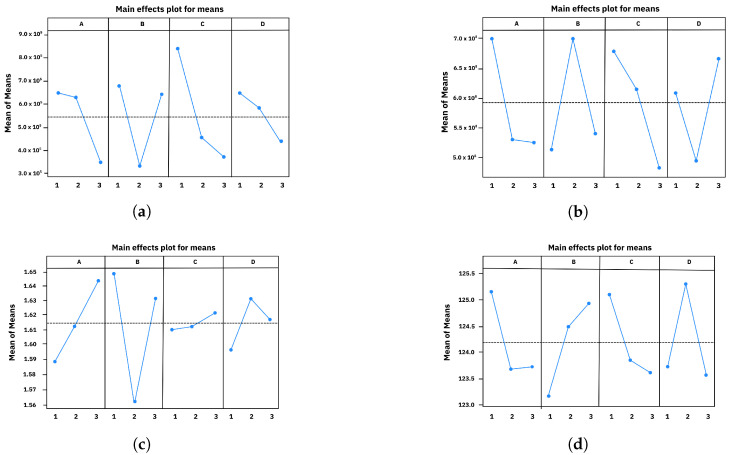
Main effect plots from Taguchi analysis for (**a**) $E_{u}$, (**b**) $E_{0}$, (**c**) loss factor (tan δ), and (**d**) glass transition temperature ($T_{g}$).

The Taguchi analysis provided further insights into the influence of the printing parameters on the dynamic mechanical properties of the ABS samples [3,24]. An L9 orthogonal array was employed to study four factors at three levels, allowing for a reduced number of experiments while focusing on the main effects of each parameter. The results of the DMA tests for this experimental design are summarized in Table 6. The main effects plots for $E_{u}$, $E_{0}$, tan δ, and $T_{g}$ (Figure 7) highlight the significance of each factor (infill pattern, build orientation, layer height, and color) in achieving the desired mechanical performance.

- *Optimal conditions for flexibility and high damping.* Conversely, for applications that require greater energy dissipation (higher tan δ) along with reduced rigidity (lower $E_{u}$, $E_{0}$ and $T_{g}$), the optimal combination would be the diamonds pattern, flat orientation, medium to high resolution (200 or 300 μm), and magenta color. These conditions are consistent with the previously discussed RSM approach, highlighting the flexibility of the material in scenarios where higher damping is desired.
- *Optimal conditions for high rigidity and low damping.* To maximize $E_{u}$, $E_{0}$, and $T_{g}$ while minimizing tan δ, the analysis indicates that the honeycomb pattern, edge orientation, resolution of 70 μm, and black color are the most suitable conditions. These results align with the RSM findings, where the honeycomb pattern and edge orientation were identified as favorable for achieving high storage compliance. The black color potentially contributes to higher $T_{g}$ due to its thermal absorption properties, promoting a more rigid material response.

The combined use of Taguchi and RSM was intentional to balance experimental efficiency with analytical depth. The Taguchi method, based on orthogonal arrays, was first applied to efficiently identify the main effects of the four printing parameters using only nine experimental runs. This approach significantly reduced the number of required samples while capturing key trends in the data. Subsequently, RSM was employed to conduct multiresponse optimization across the full experimental domain.

Together, these methods provided complementary strengths: Taguchi enabled initial screening and practical parameter selection with reduced experimental effort, while RSM offered refined predictions and visualization of trade-offs between stiffness, damping, and thermal transitions. This sequential approach enhances the robustness and applicability of the optimization strategy.

### 3.7. Comparison of Optimization Approaches

While the Taguchi method offered practical recommendations for selecting suitable parameter combinations, and the RSM provided a predictive understanding of the response trends, both methods led to consistent optimal conditions. This agreement reinforces the reliability of the proposed settings and supports their use for future processing.

- *Infill pattern.* The influence of the infill pattern on the mechanical properties of 3D-printed ABS specimens is evident in this study. The honeycomb pattern demonstrated a higher elastic modulus compared to the diamonds pattern, suggesting a more effective load distribution and better interlayer bonding [40]. This finding is consistent with previous research that highlights the importance of selecting an appropriate infill pattern to optimize mechanical performance based on the intended application [41]. While the diamonds pattern exhibited lower rigidity, it is important to note that its potential benefits may arise under different loading conditions, such as compressive or shear forces. This aligns with the findings of Liseli et al., who emphasize the need to correlate the infill pattern to the specific mechanical demands of the component [42]. Given the anisotropic nature of 3D-printed materials, optimizing the infill strategy for each application is crucial to maximizing performance. Additionally, the literature identifies the honeycomb pattern as one of the most effective strategies for enhancing tensile strength and overall mechanical performance [15]. Expanding the scope of infill patterns, considering diverse loading scenarios, would contribute to a more comprehensive understanding of the structure–property relationship in 3D-printed components.
- *Build orientation.* The mechanical behavior observed in the 3D-printed ABS specimens highlights the significant influence of build orientation on the material’s viscoelastic properties. According to the literature, specimens printed in the on-edge orientation generally exhibit the highest mechanical performance due to the most effective layer alignment relative to the load direction [9,11]. This phenomenon is consistent with the higher elastic modulus and glass transition temperature ($T_{g}$) observed in the on-edge orientation in this study. The enhanced stiffness can be attributed to the increased interlayer cohesion, which requires the crack to propagate across multiple weak interfaces before causing ultimate failure. In contrast, specimens printed in the flat orientation demonstrated lower stiffness and higher tan δ values, aligning with previous reports of reduced mechanical performance for this configuration [14]. The higher tan δ observed suggests increased energy dissipation due to interlayer delamination, further supporting the notion of lower interfacial adhesion. Specimens printed in the flat orientation present more significant interlayer defects due to greater heat dissipation during printing. The lower values of stiffness and higher energy dissipation (tan δ) observed in this study indicate a balance between adequate layer bonding and limited delamination effects. Overall, the anisotropic behavior of the ABS specimens observed here is consistent with previous research, confirming that build orientation is a critical factor in optimizing the mechanical performance of 3D-printed components [19]. The superior mechanical performance observed in the on-edge orientation, particularly in terms of resistance to deformation and energy dissipation, supports its consideration for applications demanding higher structural integrity and durability.
- *Layer height.* As a critical parameter in FDM printing, layer height significantly influences the mechanical properties of the printed components. The experimental results indicate that the best mechanical performance in terms of stiffness properties was achieved with a layer height of 70 μm. This finding aligns with previous studies that report improved interlayer adhesion and reduced porosity at lower layer heights, which result in better load distribution across adjacent layers and increased tensile strength [16]. Smaller layer heights lead to a more precise structure, minimizing voids and micro-gaps between layers. This contributes to enhanced stress transfer and improves structural integrity, especially under tensile loads. Conversely, larger layer heights, while potentially beneficial for impact resistance due to their increased material deposition, often compromise the overall surface quality and can lead to inconsistent layer bonding [35]. The literature further suggests that optimal layer height may vary depending on the specific mechanical properties desired [20]. For instance, increased layer thickness has shown favorable impact resistance, while minimal thickness favors bending strength. This balance highlights the need to tailor the layer height according to the functional requirements of the component.
- *Color.* This factor is often overlooked as influencing the mechanical properties of 3D-printed components. However, the experimental results of this study reveal that the color of ABS filaments significantly impacts the elastic behavior at high temperatures. This outcome aligns with previous research, where the mechanical performance of polymers varied with color due to differences in extrusion temperature and potential pigment interactions [17,18]. Pigments and additives used to achieve different filament colors may alter the rheological behavior of the polymer, affecting its flow characteristics and layer adhesion during the printing process [2]. Therefore, color should not be viewed merely as an aesthetic choice but as a parameter influencing the structural performance of the printed part. The colors analyzed in this study were selected to represent a range of chemical characteristics potentially affecting segmental mobility within the printed structure. The observed differences at elevated temperatures suggest that pigmentation may play a role in modifying relaxation dynamics or interlayer bonding during the printing process.

In the next section, we focus on validating these optimized conditions experimentally and integrating fractional modeling for a deeper understanding of the viscoelastic behavior across selected configurations.

### 3.8. Fractional Model Results

The viscoelastic response of the 3D-printed samples was analyzed using the FZM. While the FZM has been previously applied to conventional polymer systems [32,43,44], its implementation for additively manufactured specimens offers new insights, given their inherent anisotropy and processing-induced heterogeneity. The dynamic mechanical performance of these components is critical for applications involving thermal cycling or vibrational loads [25,28]. To quantify the influence of process parameters and filament color on the relaxation behavior, the FZM was fitted to experimental DMA data. The following sections present the estimated model parameters, the fit quality, and the interpretation of the fractional parameters across different printing configurations.

**Figure 8:**
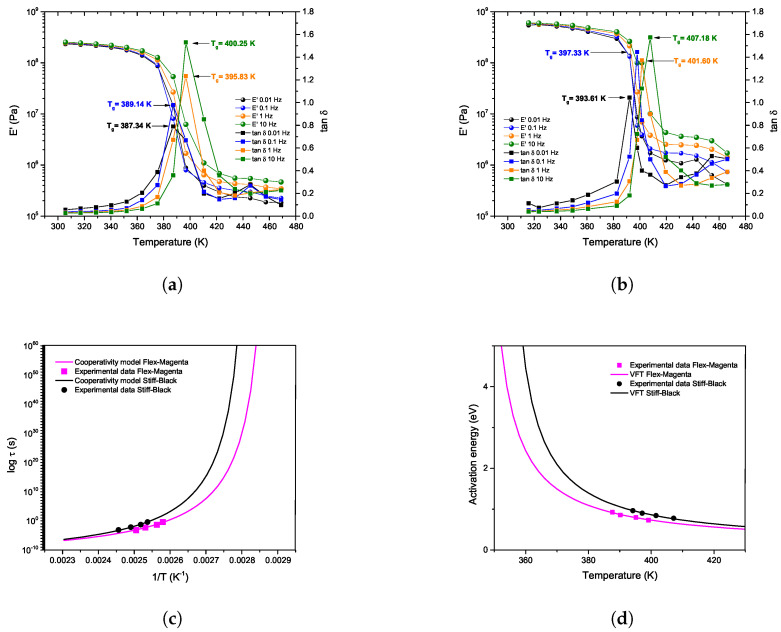
DMA and cooperative relaxation behavior: (**a**) Flex-Magenta sample, (**b**) Stiff-Black sample, (**c**) relaxation time estimation from tan δ peaks, and (**d**) activation energy vs. temperature.

In the analyzed 3D-printed samples, the FZM parameters were adjusted to match the experimental dynamic mechanical analysis (DMA) curves for both optimized 3D samples, referred to as Flex-Magenta and Stiff-Black. Figure 8a,b shows $E^{\prime }$ and tan δ curves for both 3D-printed samples. These experimental curves were obtained at constant frequencies (0.01, 0.1, 1, and 10 Hz), using a heating rate of 2 K/min. For both samples, it is clear that the glass transition peak shifts to higher temperatures as the frequency increases. Therefore, for each analyzed sample, there is a maximum in tan δ for each frequency used. From each peak in the tan δ curves, a specific relaxation time can be estimated (Figure 8c). The parameters used for $T^{*}$, $T_{0}$, $E_{a}$, and $\tau_{0}$ were defined as described in [33,45] and are shown in Table 7.

In Table 7, the magnitude of the parameters $T^{*}$ and $T_{0}$ are consistent with values reported for other polymeric materials [32,33]. The parameter $E_{a}$, which represents the activation energy of conformers that move independently (Figure 8d), also aligns with values calculated for other polymers (0.4–0.7 eV). The pre-exponential factor, $\tau_{0}$, is of the same order of magnitude as the inverse of the atomic vibration frequency. Once every FZM was calculated from the experimental curves, the theoretical isochronal curves of $E^{\prime }$ and tan δ were obtained. The resulting curves are compared with the experimental curves in the following section.

**Figure 9:**
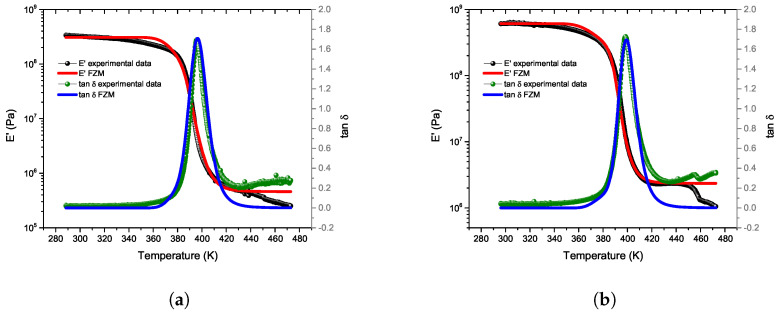
Comparison of experimental and FZM-fitted isochronal curves for (**a**) Flex-Magenta and (**b**) Stiff-Black ABS samples.

To validate the theoretical results of the FZM, a comparison with the experimental results is presented below. Table 7 and Table 8 list the FZM parameters used for the calculation of the theoretical curves shown in Figure 9. This figure presents a comparison between the theoretical and experimental results of $E^{\prime }\left(T\right)$ and tan δ for both samples, Flex-Magenta and Stiff-Black.

Figure 9 shows good agreement between the experimental isochronous spectra and the theoretical results calculated from the FZM. It can be observed that theoretical and experimental slopes of $E^{\prime }$ are similar over the temperature range analyzed. Concerning tan δ, the FZM perfectly follows the peak, even if at low temperatures and high temperatures, experimental and theoretical results did not match. This could be explained by other relaxation phenomena, at lower and higher temperatures, not being taken into account in the FZM. These deviations may be attributed to additional relaxation phenomena, such as the β-relaxation (associated with local segmental motions) at lower temperatures and viscous flow at higher temperatures. The fractional parameters α and β obtained from the FZM provide insight into the molecular relaxation mechanisms at different temperature scales.

When comparing both samples, Flex-Magenta exhibits a higher α value of 0.24, whereas Stiff-Black shows a lower value of 0.17, indicating greater molecular mobility at lower temperatures. This behavior is consistent with its lower stiffness and higher flexibility. Conversely, the Stiff-Black sample, with a lower α and higher $E_{u}$, reflects more constrained segmental motions under the same conditions. In terms of β, both samples show similar values (0.78 for Flex-Magenta and 0.76 for Stiff-Black), indicating comparable behavior in the long-time relaxation regime, typically associated with cooperative movements at higher temperatures.

When analyzing the fractional orders together with the activation energies ($E_{a}$) derived from the cooperative model (0.50 eV for Flex-Magenta and 0.52 eV for Stiff-Black), a coherent interpretation emerges in terms of molecular mobility. Flex-Magenta exhibited a higher α value (0.24) compared to Stiff-Black (0.17), which is consistent with its more elastic and less rigid nature. Since α is associated with short-time or low-temperature mobility—often linked to elastic behavior—a higher value suggests that Flex-Magenta allows easier segmental motion at these scales. On the other hand, the Stiff-Black sample, which presented a lower α and higher activation energy, shows more restricted local mobility, requiring more thermal energy to activate segmental rearrangements. The β values were similar for both samples (0.78 for Flex-Magenta and 0.76 for Stiff-Black), indicating comparable long-time viscous relaxation behavior. These results align with previous reports on amorphous and semi-crystalline polymers, where fractional orders have been related to microstructural heterogeneity and mobility constraints that vary with temperature and time scale [33,44].

This modeling approach enabled the quantification of viscoelastic behavior resulting from the combined influence of all selected FDM parameters, including pigment-related effects. The experimental design and modeling strategy allowed for an integrated assessment of structural and dynamic performance. The variations observed in fractional orders and activation energy reflect differences in relaxation behavior that are inherently linked to the sample’s build configuration, including the presence of pigment additives. The FZM results thus offer indirect yet informative indicators of interfacial quality and molecular mobility across temperature scales. In particular, the sample optimized for flexibility (Flex-Magenta) exhibited a 3.4% higher damping factor (tan δ) and a 1.46% lower glass transition temperature, while the sample optimized for stiffness (Stiff-Black) achieved an 81% increase in instantaneous modulus ($E_{u}$) and a 128% increase in equilibrium modulus ($E_{0}$). These quantitative differences reinforce the consistency between the statistical optimization and the viscoelastic modeling, confirming that the chosen parameters can be tuned to tailor mechanical behavior depending on application needs.

## 4. Conclusions

This work tackled the challenge of optimizing the viscoelastic performance of acrylonitrile butadiene styrene (ABS) parts manufactured via fused deposition modeling (FDM), focusing on how printing parameters influence mechanical and thermal behavior. To address this, a hybrid design of experiments was implemented, combining Taguchi’s L9 orthogonal array with response surface methodology (RSM) to evaluate and optimize the effects of four key factors: infill pattern, build orientation, layer height, and filament color. Build orientation and layer height emerged as the most influential parameters, significantly impacting storage modulus, damping behavior, and glass transition temperature. Edge-oriented and low-layer-height configurations increased both instantaneous and equilibrium stiffness, as well as $T_{g}$, while flat-oriented and high-resolution samples enhanced energy dissipation and flexibility. The integration of the Taguchi and RSM methods allowed efficient screening, interaction analysis, and multiresponse optimization, leading to consistent identification of optimal printing conditions. To complement the statistical findings, the viscoelastic response of two representative samples was modeled using the fractional Zener model (FZM). The model provided excellent agreement with experimental data and yielded physically meaningful parameters. The flexible sample (Flex-Magenta) exhibited a higher fractional order α=0.24, indicating increased segmental mobility at short timescales, while the stiff sample (Stiff-Black) showed a higher activation energy ($E_{a}=0.52$ eV), consistent with constrained molecular dynamics. Overall, this study demonstrates the value of integrating statistical optimization with fractional viscoelastic modeling to understand and improve the performance of 3D-printed polymers. The proposed methodology is adaptable to other materials and processing conditions and offers a generalizable framework for advancing material design in polymer additive manufacturing.

## Figures and Tables

**Table 1:** Fixed parameters used in the FDM printing of ABS samples.

Printing Parameter	Value
Filling density	100 %
Operating temperature	260 °C
Bed temperature	90 °C
Print speed	15 mm/s
Nozzle diameter	0.4 mm

**Table 2:** Experimental factors and levels considered for the statistical optimization of ABS 3D printing parameters. Letters A, B, C, and D denote the factors used in the Design of Experiments (DOE) coding.

Factor	Level		
	**1**	**2**	**3**
(A) Infill pattern	Cross	Diamonds	Honeycomb
(B) Build orientation	Flat	Upright	On-edge
(C) Layer Height	70 μm	200 μm	300 μm
(D) Filament color	White	Black	Magenta
**Mechanical response:**			
Elastic modulus at 293 K ($E_{u}$), elastic modulus at 473 K ($E_{0}$),			
loss factor (tan δ), glass transition temperature ($T_{g}$)			

**Table 3:** Experimental DMA results for ABS samples under varying printing parameters. A–D explanations refer to Table 2.

A	B	C	D	$E_{u}$ (Pa)	$E_{0}$ (Pa)	tan δ	$T_{g}$ (K)
1	2	1	3	$8.03\times 10^{8}$	$3.88\times 10^{5}$	1.5370	398.35
3	2	1	3	$8.22\times 10^{8}$	$3.25\times 10^{5}$	1.5888	397.45
1	3	2	3	$4.66\times 10^{8}$	$6.96\times 10^{5}$	1.7011	396.25
2	1	2	3	$5.50\times 10^{8}$	$5.26\times 10^{5}$	1.6475	394.85
3	1	2	3	$7.49\times 10^{8}$	$5.73\times 10^{5}$	1.6745	396.15
1	2	3	3	$4.04\times 10^{8}$	$2.53\times 10^{5}$	1.6203	396.85
2	1	3	3	$3.38\times 10^{8}$	$4.61\times 10^{5}$	1.6958	395.85
2	2	3	3	$4.06\times 10^{8}$	$2.80\times 10^{5}$	1.5280	397.25
3	3	3	3	$6.26\times 10^{8}$	$4.29\times 10^{5}$	1.6527	398.55
1	1	1	1	$7.40\times 10^{8}$	$1.17\times 10^{6}$	1.6025	397.75
2	3	1	1	$6.45\times 10^{8}$	$1.26\times 10^{6}$	1.5931	399.15
3	2	1	1	$7.14\times 10^{8}$	$4.20\times 10^{5}$	1.5798	395.45
3	3	1	1	$4.57\times 10^{8}$	$1.21\times 10^{6}$	1.6546	398.05
1	3	2	1	$5.85\times 10^{8}$	$6.62\times 10^{5}$	1.6217	398.35
2	3	2	1	$4.78\times 10^{8}$	$4.44\times 10^{5}$	1.6219	397.55
1	2	3	1	$5.89\times 10^{8}$	$3.69\times 10^{5}$	1.4813	398.75
1	3	3	1	$5.26\times 10^{8}$	$4.26\times 10^{5}$	1.5553	397.65
2	3	3	1	$3.48\times 10^{8}$	$4.85\times 10^{5}$	1.5583	400.45
1	2	1	2	$6.81\times 10^{8}$	$3.82\times 10^{5}$	1.5704	395.45
2	3	1	2	$4.77\times 10^{8}$	$1.06\times 10^{6}$	1.6399	399.55
1	1	2	2	$5.65\times 10^{8}$	$7.04\times 10^{5}$	1.4672	398.35
2	2	2	2	$6.62\times 10^{8}$	$3.25\times 10^{5}$	1.5115	399.15
3	1	2	2	$8.80\times 10^{8}$	$7.36\times 10^{5}$	1.5281	399.25
2	1	3	2	$2.08\times 10^{8}$	$3.17\times 10^{5}$	1.6476	395.75
3	3	3	2	$5.23\times 10^{8}$	$5.04\times 10^{5}$	1.6620	398.25
3	2	3	2	$3.51\times 10^{8}$	$2.89\times 10^{5}$	1.5590	397.05
3	1	3	2	$2.40\times 10^{8}$	$3.15\times 10^{5}$	1.7034	396.35

**Table 4:** MANOVA results for the effect of printing parameters on viscoelastic properties of ABS.

Factor	Statistic	Value	Num DF	Den DF	*F* Value	Pr>F
Infill pattern	Wilks’ lambda	0.6579	2	25	6.4989	0.0053
	Pillai’s trace	0.3578	2	25	6.9630	0.0039
	Hotelling–Lawley trace	0.4961	2	25	6.2009	0.0065
	Roy’s greatest root	0.4421	2	25	5.5268	0.0103
Build orientation	Wilks’ lambda	0.5095	2	25	12.0346	0.0002
	Pillai’s trace	0.5315	2	25	14.1829	0.0001
	Hotelling–Lawley trace	0.8823	2	25	11.0282	0.0004
	Roy’s greatest root	0.7789	2	25	9.7362	0.0007
Layer height	Wilks’ lambda	0.5493	2	25	10.2566	0.0006
	Pillai’s trace	0.4592	2	25	10.6157	0.0005
	Hotelling–Lawley trace	0.8050	2	25	10.0624	0.0006
	Roy’s greatest root	0.7852	2	25	9.8151	0.0007
Color	Wilks’ lambda	0.7549	2	25	4.0577	0.0298
	Pillai’s trace	0.2562	2	25	4.3046	0.0247
	Hotelling–Lawley trace	0.3099	2	25	3.8740	0.0342
	Roy’s greatest root	0.2515	2	25	3.1438	0.0605

**Table 6:** DMA response data from Taguchi L9 orthogonal array for ABS samples. A–D explanations refer to Table 2.

A	B	C	D	$E_{u}$ (Pa)	$E_{0}$ (Pa)	tan δ	$T_{g}$ (K)
1	1	1	1	$7.40\times 10^{8}$	$1.17\times 10^{6}$	1.6025	397.75
1	2	2	2	$7.53\times 10^{8}$	$3.66\times 10^{5}$	1.5519	399.35
1	3	3	3	$6.28\times 10^{8}$	$4.36\times 10^{5}$	1.6140	397.85
2	1	2	3	$5.50\times 10^{8}$	$5.26\times 10^{5}$	1.6475	394.85
2	2	3	1	$5.56\times 10^{8}$	$3.35\times 10^{5}$	1.5499	396.05
2	3	1	2	$4.77\times 10^{8}$	$1.06\times 10^{6}$	1.6399	399.55
3	1	3	2	$2.40\times 10^{8}$	$3.15\times 10^{5}$	1.7034	396.35
3	2	1	3	$8.22\times 10^{8}$	$3.25\times 10^{5}$	1.5888	397.45
3	3	2	1	$5.16\times 10^{8}$	$4.23\times 10^{5}$	1.6390	396.75

**Table 7:** Parameters used in the cooperative relaxation time model for Flex-Magenta and Stiff-Black samples.

Sample	$T^{*}$ (K)	$T_{0}$ (K)	$E_{a}$ (eV)	$\tau_{0}$ (s)
Flex-Magenta	432	345	0.50	$4\times 10^{-13}$
Stiff-Black	440	352	0.52	$2\times 10^{-13}$

$T^{*}$: onset temperature of cooperative motion; $T_{0}$: Vogel temperature; $E_{a}$: activation energy; $\tau_{0}$: pre-exponential factor of the relaxation time.

**Table 8:** Fractional Zener model parameters fitted to experimental DMA data of ABS samples.

Sample	$E_{U}$ (Pa)	$E_{0}$ (Pa)	α	β
Flex-Magenta	$3.37\times 10^{8}$	$4.61\times 10^{5}$	0.24	0.78
Stiff-Black	$6.11\times 10^{8}$	$1.05\times 10^{6}$	0.17	0.76

$E_{u}$: elastic modulus at low temperatures; $E_{0}$: elastic modulus at high temperatures; α, β: fractional orders describing the viscoelastic response.

## Data Availability

The original contributions presented in this study are included in the article. Further inquiries can be directed to the corresponding authors.
